# Cancer-associated Macrophage-like Cells in Patients with Non-metastatic Adenocarcinoma of the Esophagus - Cytomorphological Heterogeneity

**DOI:** 10.7150/jca.82668

**Published:** 2023-07-09

**Authors:** Clara Braun, Claudia Schmoor, Sylvia Timme-Bronsert, Stefan Fichtner-Feigl, Jens Hoeppner, Birte Kulemann, Jasmina Kuvendjiska

**Affiliations:** 1 Faculty of Medicine, Albert-Ludwigs-University of Freiburg, Germany.; 2 Clinical Trials Unit, Faculty of Medicine and Medical Center, University of Freiburg, Germany.; 3 Tumorbank, Comprehensive Cancer Center Freiburg, University Medical Center Freiburg, Germany.; 4 Institute for Surgical Pathology, University Medical Center Freiburg, Germany.; 5 Department of General and Visceral Surgery, University Medical Center Freiburg, Germany.; 6 Department of Surgery, University Medical Center Schleswig-Holstein, Lübeck, Germany.

**Keywords:** cancer-associated macrophage-like cell, CAML, esophageal adenocarcinoma, cytopathology, liquid biopsy, liquid biomarker

## Abstract

**Introduction:** Esophageal adenocarcinoma (EAC) often recurs systemically despite therapy with a curative aim. New diagnostic and therapeutic approaches are urgently needed. A promising field is liquid biopsy, meaning the investigation of tumor-associated cells in the peripheral blood, for example cancer-associated macrophage-like cells (CAML). The aim of this multicentric study was to investigate the presence and cytomorphological appearance of CAML in patients with non-metastatic and operable esophageal cancer.

**Methods:** Blood samples from 252 patients with locally advanced EAC were obtained before starting curative treatment including surgery, and then processed using ScreenCell® filtration devices. Cytological analysis was performed via May-Grünwald-Giemsa staining. CAML were defined by their morphological characteristics. We also performed immunofluorescence staining with the mesenchymal marker vimentin on a subset of our study cohort.

**Results:** We detected cytomorphologically heterogeneous CAML in 31.8% (n=80) patients. Their presence and cell count did not correlate significantly with pretherapeutic cTNM. Even in patients with small tumors and no lymph-node infiltration, cell counts were high. CAML showed heterogenous staining patterns for vimentin.

**Conclusion:** This is one of the first studies demonstrating the presence and phenotype of CAML in a uniquely broad cohort of EAC patients. As they are believed to be representatives of the inflammatory tumor microenvironment shed into the bloodstream, their presence in non-metastatic EAC is a promising finding.

## Introduction

Esophageal carcinoma is the world's seventh most common cancer and the sixth most common cause of cancer death worldwide 1. Esophageal adenocarcinoma (EAC) is a particular problem in the western world due to its increasing incidence and still poor prognosis despite multimodal therapy approaches 2,3. EAC is usually diagnosed at already advanced stages because of its unspecific clinical presentation 4. When there is no clinical sign of metastasis, patients undergo neoadjuvant treatment and esophageal resection 5. Unfortunately, around 50% of patients who undergo an initially curatively-intended resection of a clinically non-metastatic primary tumor develop distant metastases 6-10. Diagnosis and follow-up are currently carried out via computer tomography and endoscopy, but there are no sensitive and specific tumor markers 11,12. Since metastases develop so often in curatively treated patients 7, we must assume that micro-metastatic disease is being missed by the current staging tools - potentially resulting in unnecessary surgery. We therefore urgently need novel diagnostic tools that can detect metastatic disease and assess the treatment response or early progression in EAC patients 3,13.

Liquid biopsy offers great potential in helping us understand tumor biology and metastasis better. Circulating tumor cells (CTC) are the oldest and most well-known liquid biomarkers 14. In the case of esophageal cancer, the presence of CTC determined by the CellSearch® system correlates with poor prognosis 15. We already reported the presence of CTC detected by ScreenCell® in patients with EAC during the course of multimodal treatment 16. As the CTC detection rate is low in EAC, especially at operable, non-metastatic stages 17,18 we urgently need other liquid biomarkers.

With increasing knowledge about the complex interaction between tumorous and body tissue, other tumor-associated cells originating from the tumor microenvironment have recently come into focus in addition to CTC, for example cancer-associated macrophage-like cells (CAML) 19,20. These are believed to be disseminated tumor-associated macrophages (TAM) that internalize tumor debris at the primary tumor site and then spread into bloodstream 19,21. Other authors have even claimed that CAML may be a fusion product between tumor cells and TAM 22,23. CAML have been identified in patients with many different entities of solid tumors, but not in healthy individuals 19,21,24-26. The first studies examining their clinical significance delivered promising results: In patients with metastatic breast cancer, the presence of CAML before treatment was associated with worse progression-free survival (PFS) and overall survival (OS) 27. Furthermore, the presence of CAML ≥50 µm after completing chemoradiotherapy in patients with Non-Small-Cell Lung Cancer (NSCLC) was associated with developing metastatic disease and worse survival 28.

In EAC terms, there are only few reports on the existence of CAML. In a recently published paper on CAML in EC patients (EAC and SCC), the authors reported superior 2-year PFS and OS in patients with CAML < 50 μm after chemoradiation compared to patients with CAML ≥ 50 μm 17. Although these initial results relied on a small and mixed patient collective, they nevertheless reveal the potential “predictive marker” quality of CAML in EAC patients - a finding justifying further investigations.

The aim of this study was to assess the presence and cytomorphological appearance of CAML in a large collective of therapy-naive patients suffering from locally advanced, non-metastatic EAC.

## Materials and Methods

### Patient selection

We enrolled 252 patients from 18 centers all over Germany (Freiburg, Magdeburg, Würzburg, Münster, Aachen, Leipzig, Mainz, München (LMU), Hamburg Eppendorf, Dresden, Offenbach, Berlin (CVK), Minden, Köln, Berlin (CBF), Dortmund, Erlangen, Stuttgart RBK) between 2016 and 2020. Our inclusion criteria were: (a) histologically confirmed esophageal adenocarcinoma according to UICC TNM7 definition 29 or Siewert classification AEG type 1 and type 2 or 3 in case of esophageal infiltration. (b) Pretherapeutic stage cT1 N+ M0 or cT2-4a N0/N+ M0. (c) No prior chemotherapy for gastrointestinal cancer, and no prior abdominal or thoracic radiotherapy. Patients with esophageal tumors other than adenocarcinoma were excluded, as were patients with metastatic tumors or not curatively-resectable tumors. The clinical staging was assessed identically in all study centres. All patients underwent thoracic and abdominal CT scans, gastro-esophageal endoscopy and endosonography. All patients gave full informed consent for material, data acquisition and the following experiments. This study was approved by the Ethics Committee of Albert-Ludwigs-University Freiburg (315/15 FF-MC), Freiburg, Germany.

### Cell analysis

To detect CAML, peripheral venous blood samples were taken before initiating any neoadjuvant treatment. Blood specimens were collected using transfix tubes (Circulating Tumor Cell TransFix/EDTA Vacuum Blood Collection Tubes 9ml, Cytomark, Caltag Medsystems Ltd, Buckingham, UK). The tubes were sent within 24 hours after blood draw to the CTC laboratory at the Department of Surgery, University Medical Center Freiburg.

**Figure 1:**
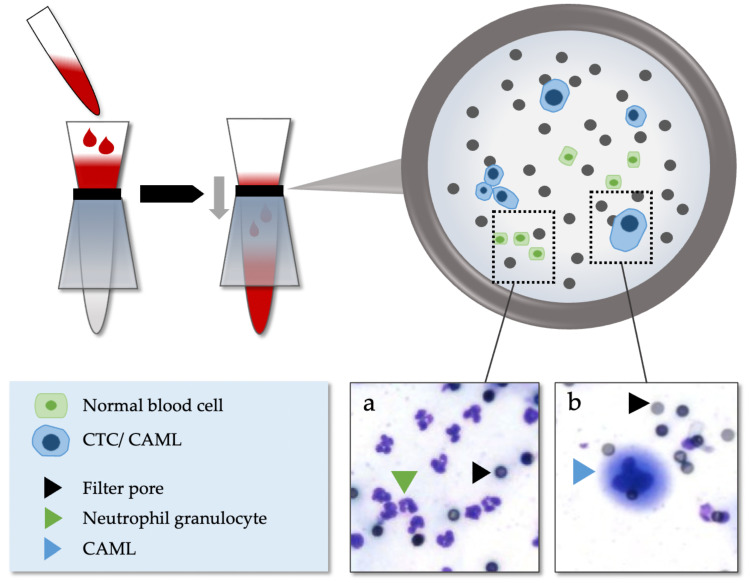
Isolation method of CAML and CTC via ScreenCell®. (a) Normal blood cells. (b) Example of CAML in patients with EAC.

After 4 hours to 5 days of storage at room temperature, the blood specimens were processed using ScreenCell® Cyto-R devices (ScreenCell, Sarcelles, France) according to the manufacturer's instructions and as reported previously 16,30,31. ScreenCell® is a surface marker independent enrichment technology using microfilters through which almost all red and white blood cells are removed via low-pressure vacuum-filtration. Enlarged cells remain on the filter membrane and can subsequently be stained and analyzed cytologically (Figure 1). At least one filter was created for every patient using 3 ml of blood for each filter.

After being processed with the ScreenCell® device, the filters were first stored at 4°C and later at minus 20°C, as it became clear to us that storage at minus 20°C was yielding better staining results. For staining, filters were dried at 38°C for 60 minutes. They were stained with standard May-Grünwald-Giemsa-staining for cytological analysis. Stained filters were analyzed by two trained readers blinded to the diagnosis of the patients on brightfield using an Olympus BX16 microscope. CAML were identified by their cytomorphological characteristics as described before 17,19,27,28, which are (a) extraordinary large cells with a relatively low nucleo-cytoplasmic ratio, (b) enlarged multilobulated nuclei, and (c) voluminous cytoplasm. Every CAML was photographed and documented. Questionable interpretations were re-evaluated until a consensus was reached and analyzed by a cytopathologist for verification.

### Statistics

All statistical analyses were performed using SAS/STAT Software Version 9.3 (SAS Institute Inc., Cary, NC, USA). No formal sample size calculation was done. Data were analyzed descriptively. Categorial data were summarized by absolute and relative frequencies. Continuous data were summarized by mean, standard deviation, median, quartiles and range. The association between CAML and clinical T- and N-stage was compared using chi-square tests at a two-sided significance level alpha of 5%. Therefore, the probability of any CAML was compared between patients with small tumors (cT1-2) versus large tumors (cT3-4) and between patients with cN0 versus cN+. The probability of any CAML was estimated with 95% confidence intervals in the whole study population and in subgroups defined by cT and cN. P-values of p<0.05 were considered significant.

### Immunofluorescence staining

For some patients presenting CAML on the first filter in Giemsa staining, we had access to a second ScreenCell® filter for immunofluorescence staining.

Before staining, filters that had been stored at minus 20°C were dried at 38°C for 60 minutes or left to dry at room temperature overnight. Then they were washed with 500µl of Phosphat buffered saline (PBS) for 5 minutes. Cell membranes were lysed with 500µl of permeabilization buffer (PBS.T, 0.5% Triton in PBS, Sigma-Aldrich, Merck, Germany). Filters were washed again three times with 500µl of PBS for 5 minutes and blocked with 500µl of normal goat serum (NGS, 2% in PBS) for 30 minutes. Afterwards, they were incubated with anti-vimentin monoclonal antibody (rabbit, GTX 16700, GeneTex, Irvine, USA; diluted 1:1000 in 2% NGS) overnight at 4°C in a humidity chamber. Filters were washed again four times with 500µl PBS for 5 minutes and incubated with the secondary antibody anti-rabbit Cy3 (A-10520, ThermoFisher Scientific Inc., Waltham, USA; diluted 1:1000 in 2% NGS) for 60 minutes at room temperature. Then they were washed twice with 500µl PBS for 5 minutes. For nuclear staining, Hoechst 33342 (ThermoFisher Scientific Inc., Waltham, USA; diluted 1:1000 in H_2_O) was added for 3 minutes and washed again twice with PBS for 5 minutes and then H_2_O. After drying at room temperature, the filters were analyzed using Olympus BX61 microscope and suspicious cells were photographed.

To verify the suspicious cells, standard hemotoxylin-eosin (HE) staining was performed subsequently using Hemacolor rapid staining kit (Sigma-Aldrich, Merck, Darmstadt, Germany) without having washed off the immunofluorescence stain. Hemacolor solutions were filtered before usage to prevent impurity with large staining particles. Cells that had been photographed in immunofluorescence staining were subjected to brightfield microscopy and photographed again.

Only filters stored at minus 20°C were included for our immunofluorescence assessments as this resulted in better staining results than storage at 4°C.

## Results

### Study population

Our study cohort contained 252 patients. Patient characteristics are illustrated in Table 1. 88.5% (n=223/252) of patients were male. Their average age was 63 years. Most had a tumor at clinical T3 stage (74.6%, n=188/252). Lymph node infiltration (cN+) was detectable in 79% (n=199/252) during pre-therapeutic imaging. No patient showed clinical signs of distant metastasis (cM0).

### CAML

#### Cytomorphological characterization of CAML

**Figure 2:**
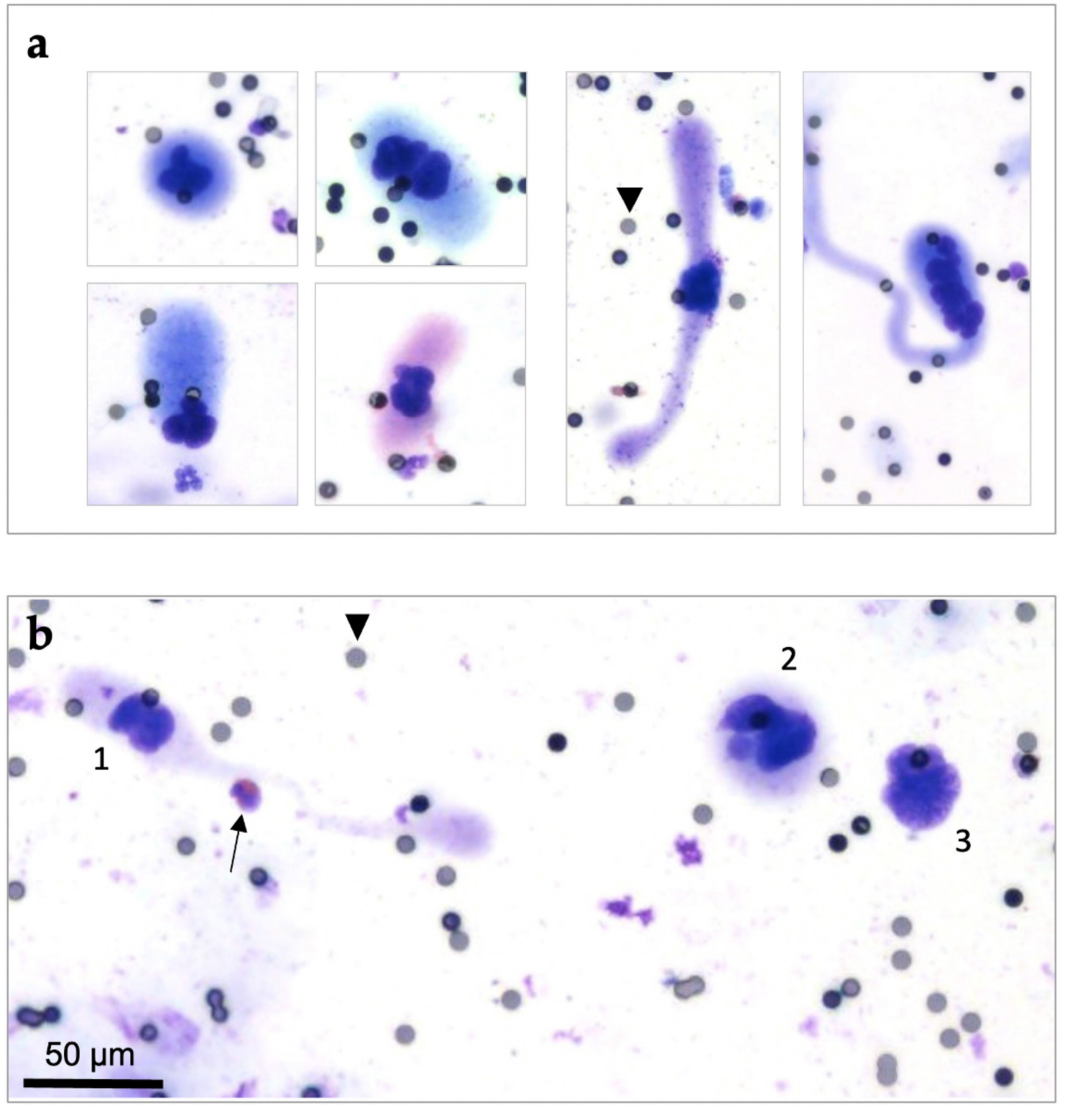
Cytomorphological heterogeneity of CAML on ScreenCell® filters, Giemsa staining (20x magnified). Pores are exemplarily marked by black arrowheads (pore size: 7,5µm). **(a)** Different cytoplasmic and nuclear configurations of CAML. Left: Smaller CAML with round or oval shaped cytoplasm. Right: Large CAML with long cytoplasmic “tails”. **(b)** Cytological comparison between CAML (1 and 2) versus CTC (3) and eosinophil granulocyte (marked with black arrow). CAML were defined as extraordinary large cells with a relatively low nucleo-cytoplasmic ratio, enlarged multilobulated nuclei and voluminous cytoplasm. CTC were defined as cells with enlarged (≥ 16µm) and hyperchromatic nuclei with irregular nuclear borders and increased nucleo-cytoplasmic ratio.

CAML were highly heterogenous in terms of size range, nuclear profile, and cytoplasmic configuration, even in a single patient. Some CAML were quite small and round or oval shaped. Other CAML were remarkably large (up to more than 100 µm), rod shaped, or revealing a wide variety of cytoplasm “tails” (Figure 2). All CAML were much larger than normal blood cells (Figure 2b). CAML tended to exhibit multilobulated nuclei, and some even had several separate nuclei.

#### Quantity of CAML and Correlation with TNM

A minimum of one CAML per filter (3 ml of patient blood) was detected in 31.8% (n=80/252) of patients (CI: 26.1%; 37.9%). The mean number from the entire study cohort was 2.7 CAML per filter (Table 2). When patients were positive for CAML, the cell count was rather high: In CAML-positive patients, the mean number was 8.4 cells per filter. The range here was very high: while most patients had no CAML at all, some had up to 47 CAML on one filter.

The CAML count showed no association with the clinical T- and N-stages (Table 2). The highest CAML number per filter (n=47) was identified in a patient suffering from advanced local spread (cT3) and lymph node infiltration (cN+), whereas the second highest CAML count (n=44) was found in a patient presenting a smaller local tumor stage (cT2) and no signs of lymph node infiltration (cN0).

CAML-positivity, regardless of the cell count, also failed to demonstrate any significant association with the tumor stage (Table 3) (T1-2: 36.0%, 95%CI=22.9-50.8% versus T3-4: 30.7%, 95%CI=24.4-37.6%, p=0.4704), nor with lymph node invasion (N0: 39.6%, 95% KI=26.4-54.0% versus N+: 29.7%, 95% KI=23.4-36.6%, p=0.17).

In addition to CAML, we detected CTC in 65.5% (n=165/252) of patients. To clarify the difference between CAML, CTC, and normal blood cells, Figure 2b illustrates them all in one diagram. The defining criteria we used can be found in detail in our previous publication 31.

#### Immunofluorescence staining

**Figure 3:**
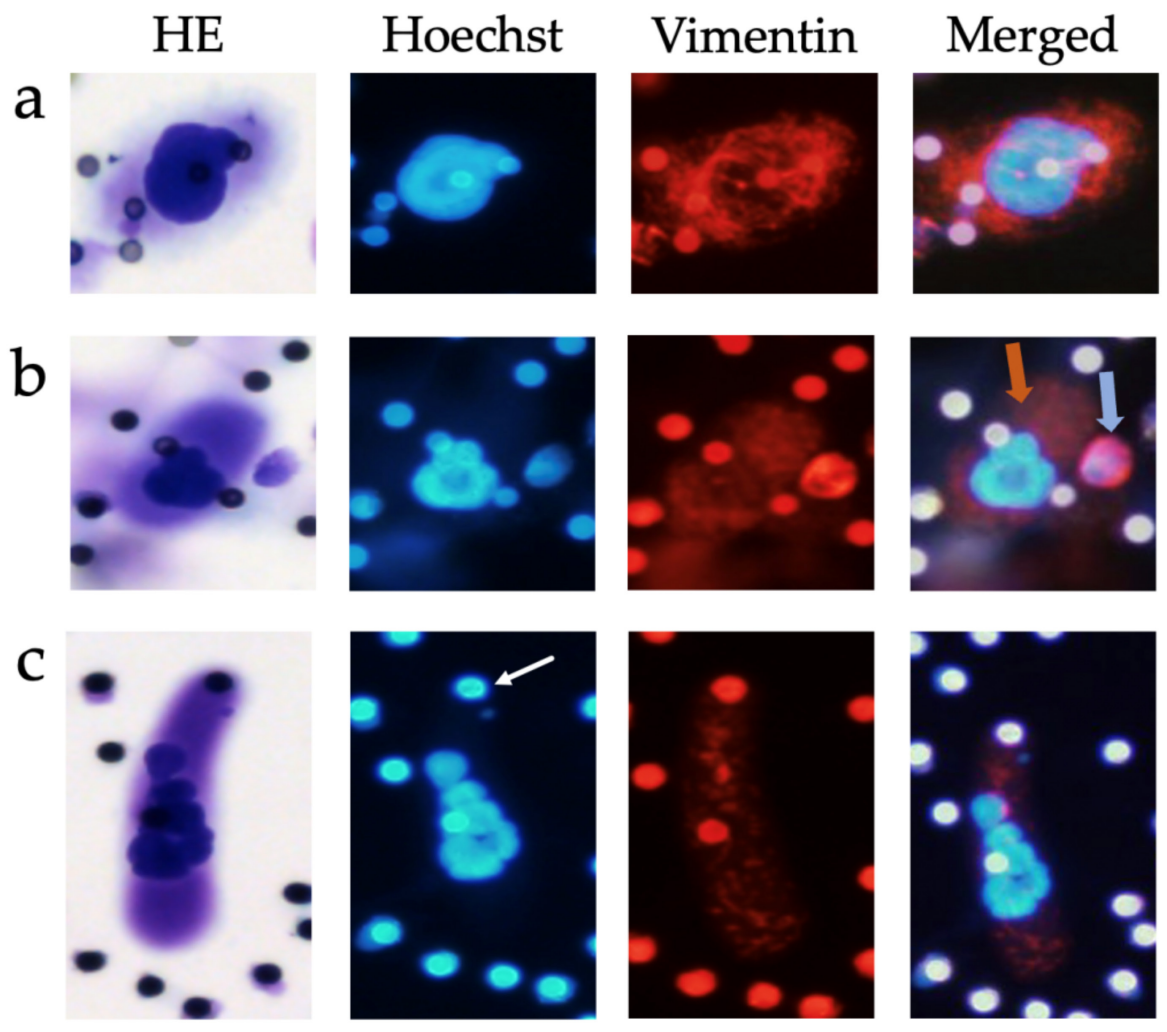
Examples of CAML immunofluorescence staining, 20x magnified. **Row (a)** CAML with strong vimentin-positive cytoplasm. **Row (b)** CAML with moderate positive cytoplasm (orange arrow) next to a leukocyte with strong vimentin-positive cytoplasm (blue arrow). **Row (c)** CAML with moderate vimentin-positive cytoplasm. Pores appeared auto-immunofluorescent, highlighted here with a white arrow (pore size 7.5µm). HE: hemotoxylin-eosin staining

CAML revealed heterogeneous cytoplasmic staining patterns in conjunction with the mesenchymal marker vimentin. Vimentin is expressed by leukocytes, but also by cancer cells following epithelial-mesenchymal transition (EMT), which is why it is commonly used as marker for mesenchymal phenotypes in EMT studies 32. Most CAML expressed vimentin, but the intensity of marker expression varied between no expression at all to very strong vimentin signals (Figure 3), even in the same patient. One exemplary patient at stage cT4cN+cM0, who presented 12 CAML on the first Giemsa staining filter, showed 10 CAML on the second filter. Of those CAML, five exhibited no or very weak vimentin expression, whereas the other five CAML were moderately to strongly vimentin-positive. CAMLs' vimentin staining patterns were usually inhomogeneous and punctual, or filamentous compared to the leukocytes' staining pattern (Figure 3b) or that of CTC-Clusters, which also stained positive for vimentin. Single-cell CTC never stained positive for vimentin in our study participants.

## Discussion

This multicentric study demonstrates the presence and cytomorphological features of CAML in patients diagnosed with locally advanced esophageal adenocarcinoma (EAC) prior to multimodal treatment including surgery. Since esophageal cancer is a comparatively rare tumor entity 33 our study cohort's large size (over 250 patients) is particularly valuable and unique. Our patients enable initial insights into the presence of a promising new liquid biopsy marker in a tumor entity that because of its poor prognosis urgently requires new diagnostic approaches.

Most of our patients were male (n=223/252, 88.5%) which is consistent with the known gender distribution in EAC. The patients' tumors were mostly at locally advanced stages: 74.4% showed extensive local infiltration (cT3) and 79% had positive local lymph node infiltration (N+) (Table 1). Importantly, although no patient presented any signs of distant metastasis, we detected CAML in the peripheral blood samples in 31.8% (n=80) of patients.

Interestingly, the presence and quantity of CAML displayed no association with the clinical TNM stage. CAML were also observed in conjunction with small local tumors and in patients without lymph-node infiltration (N0). This evidence might indicate early, systemic spread of tumor-associated cells that go undetected via conventional diagnostic methods. Other authors have also described detecting CAML at early cancer stages, for example in breast 19,26, pancreatic 19 and esophageal cancer 17.

Little is known about the clinical significance of CAML, especially in EAC. Recently, Gironda et al. published a prospective pilot study of 32 patients with locally advanced esophageal cancer, including ESCC and EAC, in which they described the sequential presence of CAML during chemoradiotherapy 17. CAML were identified using CellSieve microfilters in 88% of all patient samples (n=28/32) and in 76% of patient samples prior to therapy (n=22/29). Interestingly, CAML size ≥ 50 μm at the completion of chemoradiotherapy was associated with poorer PFS (HR=12.0; 95%CI=2.7-54.1; p=0.004) and OS (HR=9.0; 95%CI=1.9-43.5; p=0.019). These results might demonstrate the potential of relying on CAML in disease surveillance to identify more aggressive EC subtypes and monitor the treatment response 17.

In the aforementioned study 76% of patient samples prior to therapy (n=22/29) showed at least one CAML 17 whereas in our cohort, only 31.7% of samples (n=80/252) were CAML-positive before neoadjuvant treatment. This discrepancy could be attributable to differences in study design and patient selection: Our study included only EAC patients, while Gironda et al. also included ESCC patients. Moreover, their patients received chemoradiation, whereas in our cohorts, all tumors were considered operable, and all patients were scheduled for multimodal treatment including surgery. Employing different detection methods can also affect detection rates.

Recent studies measuring CAML size reinforce the theory that larger CAML correlate with more aggressive disease: the presence of CAML ≥50μm is considered a predictor of poor prognosis 21. In our study, precise size measurements were not feasible which represents a limitation of our study design.

There is to date neither a standardized definition nor specific markers for CAML 27. Other authors defined CAML by a combination of cytomorphological appearance and marker expression 19,24-26,34. Due to their large size and heterogeneous marker expression profile, size-based enrichment technologies are regarded as the gold standard for CAML detection 20,21. CellSieve® microfilters were used in most studies. CellSieve® is a filtration-based enrichment technology using immunofluorescence staining with cytokeratin, CD45 and DAPI to identify cells 19,34. Since the main distinguishing feature of CAML lies in their phenotype, and this phenotype is also visible in brightfield microscopy, we do not believe that immunofluorescence staining must be used to identify CAML. We therefore used ScreenCell®, which is also a filtration-based CTC enrichment technology 31,35,36 that our group already used in other CTC studies 16,30,36. It preserves cytomorphological cell features, thus allowing cytomorphological analysis. The ScreenCell® Cyto device also enabled our multicentric study design, as it allowed the transportation and time-delayed processing of blood samples.

To gain more insights into the cellular characteristics and EMT status of CAML, we conducted immunofluorescence staining with the mesenchymal marker vimentin in patients from whom a second filter was available and who showed CAML on the first filter in Giemsa staining. CAML revealed heterogeneous staining patterns for vimentin. This finding is consistent with previous reports of CAML expressing a broad spectrum of markers at highly variable levels, including epithelial, macrophage, and endothelial markers 19,20,26,37. The rather inhomogeneous and punctual staining pattern for vimentin may be attributable to phagocytic tumor material from the tumor site. This theory of ingested tumor debris has been reinforced by other investigators who demonstrated tumor-specific marker expression (for example PDX-1 for pancreatic cancer) within CAML 19. CAMLs' varying differentiation stages is another potential explanation for heterogenous staining patterns 38, as are variations in transportation and storage due to our multicentric study design.

The heterogeneity in marker expression and phenotype makes investigating CAML properties in terms of single-cell analysis more challenging. Future investigation is needed to clarify their marker expression profile. Nevertheless, the latest studies indicate that using CAML as biomarker improves the clinical application of cell-based liquid biopsy 17,21,23,28,39. Because of their heterogeneous appearance, filtration-based enrichment technologies are particularly well-suited for gaining initial insights into the occurrence of CAML in cancer patients.

CAML are believed to be representatives of the local inflammatory tumor microenvironment 28. Inflammation plays a major role in tumor progression and metastasis 40. The detection of possible biomarkers for the interplay between tumor and immune system is thus of great interest, especially in a tumor entity like EAC which is known to be a paradigm of inflammation-induced cancer through its association with gastroesophageal reflux 13,41.

Our study illustrates the presence and heterogeneity of CAML in a uniquely broad cohort of therapy-naive EAC patients. Showing the fact, that CAML occur also in patients with localized tumor stages might support others establishing effective CAML isolation protocols; thus our data might pave the way for deeper analysis of genetic and molecular features of CAML in EAC. CAML surveillance over treatment time, like the CTC-analyses we already published in a pilot study 16, is planned and will be published when follow-up data are available.

## Conclusion

CAML are present in patients with non-metastatic, locally advanced esophageal adenocarcinoma (EAC). They exhibit heterogeneous cytomorphological features in terms of size, cytoplasmic configuration, nuclear shape and marker expression. The role of CAML as a predictive marker in EAC is yet to be determined.

## Figures and Tables

**Table 1:** Patient characteristics and TNM stage

Number of patients (n)	252
Gender (male/female), n (%)	223 (88.5%)/ 29 (11.5%)
Age in years, mean (range)	63.0 (37-86)
BMI in kg/ m², mean (range)	27.5 (14.5-57.1)
Clinical TNM stage, n (%)	
cT1	3 (1.2)
cT2	47 (18.7)
cT3	188 (74.6)
cT4	14 (5.6)
cN0	53 (21.0)
cN+	199 (79.0)
cM0	252 (100.0)

cT-Stage: size of primary tumor; cN-Stage: degree of spread in regional lymph nodes; cM-Stage: presence of distant metastases; BMI: body mass index

**Table 2:** Quantity of CAML per filter (3ml of patient blood) associated with tumor stage

		n	Mean	SD	Minimum	Lower Quartile	Median	Upper Quartile	Maximum
**Total**		252	2.7	6.977	0	0	0	1	47
	**cT-stage**								
	T1-2	50	3.0	7.941	0	0	0	2	44
	T3-4	202	2.6	6.736	0	0	0	1	47
	**cN-stage**								
	N0	53	3.2	7.376	0	0	0	2	44
	N+	199	2.5	6.878	0	0	0	1	47
**CAML-positive patients**		80	8.4	10.318	1	1	4	10.5	47
	**cT -stage**								
	T1-2	18	8.3	11.606	1	1	4	9	44
	T3-4	62	8.4	10.017	1	1	4	11	47
	**cN-stage**								
	N0	21	8.1	9.995	1	2	4	9	44
	N+	59	8.4	10.513	1	1	4	12	47

cT-Stage: size of pimary tumor, cN-Stage: degree of spread in regional lymph nodes, n: number of patients, SD: standard deviation. The upper section includes all patients, meaning samples with CAML and without CAML. The lower section shows only patients with at least one CAML per filter.

**Table 3:** Association of CAML-positivity and clinical TNM-stage.

	n	n (CAML positive)	%	CI	p
**cT-stage**					
T1-2	50	18	36.0	22.9%; 50.8%	0.47
T3-4	202	62	30.7	24.4%; 37.6%	
**cN-stage**					
N0	53	21	39.6	26.5%; 54,0%	0.17
N+	199	59	29.7	23.4%; 36.5%	

cT-Stage: size of primary tumor, cN-Stage: degree of spread in regional lymph nodes, n: number of patients, CI: confidence interval, p: p-value

## Abbreviations

CAML: cancer-associated macrophage-like cells
CTC: circulating tumor cells
EAC: esophageal adenocarcinoma
EC: esophageal cancer
ESCC: esophageal squamous cell carcinoma
EMT: epithelial-mesenchymal transition
HE: hemotoxylin-eosin staining
TAM: tumor-associated macrophage
